# Efficacy of sub-Tenon's block using an equal volume of local anaesthetic administered either as a single or as divided doses. A randomised clinical trial

**DOI:** 10.1186/1471-2253-9-2

**Published:** 2009-03-26

**Authors:** Ehtesham I Khan, Jawad Mustafa, John McAdoo, George Shorten

**Affiliations:** 1 Dept of Anaesthesia, Cork University Hospital and University College Cork, Ireland, Wilton, Cork, Co Cork, Ireland

## Abstract

**Background:**

Sub-Tenon's anaesthetic is effective and reliable in producing both akinesia and anaesthesia for cataract surgery. Our clinical experience indicates that it is sometimes necessary when absolute akinesia is required during surgery to augment the block with 1–2 ml of local anaesthetic. Hypothesis was that after first injection some of the volume injected may spill out and before second injection the effect of hyaluronidase has taken place and second volume injectate will have desired effect.

**Methods:**

A prospective, randomised, control trial in which patients were randomly allocated to one of two groups. In group 1, single injection of 5 ml of local anaesthetic was injected. In group 2, 3 ml of the same anaesthetic solution was injected followed by application of gentle orbital pressure for 2 minutes. A further 2 ml of the same anaesthetic solution was injected through the same conjunctival incision. Measurement of movement in four quadrants of eye was done by the surgeon at 3 and 6 minutes. Intraocular pressure, chemosis, and subconjuctival haemorrhage were also measured.

**Results:**

Significant differences at 3 minutes between groups for overall movement, medial, superior, and lateral quadrants occurred. At 6 minutes no significant group differences emerged for the overall movement or for any of four quadrants.

**Conclusion:**

Single injection of local anaesthesia for sub-Tenon's block with mixture of lignocaine with adrenaline, bupivacaine and hyaluronidase was found to be superior to provide akinesia of ocular muscles compared to divided dose given by two injections. No difference in groups in terms of haemorrhage, chemosis, patient's satisfaction and intraocular pressure was found.

**Trial registration:**

Trial registration no-ISRCTN73431052

## Background

Local or regional neural blockade is the preferred anaesthetic technique for cataract surgery[1]. A regional anaesthetic technique such as retrobulbar, peribulbar or sub-Tenon's block can provide ocular akinesia in addition to anaesthesia. In 1884 Knapp first described retro bulbar anaesthesia [2]. It became the preferred anaesthesia technique for ophthalmic surgery. Rare but serious complications (globe perforation, brain stem anaesthesia, retrobulbar haematoma, postoperative strabismus, optic nerve damage) led to decline in its use as clinicians tended to employ a peribulbar technique[3]. However peribulbar blockade has some limitations. First it does not eliminate serious complications; second, even with two injections there were high rate of imperfect block[3]. In 1992 Stevens described a technique for sub-Tenon's anaesthesia, which entailed application of topical anaesthesia, use of an eye speculum, making a small incision in the conjunctiva, and passing a blunt cannula posteriorly in the subconjuctival space[4]. Injectate administrated at this site passes into the sub-Tenon's space. Since 1992 many studies support sub-Tenon's block as a relatively safe technique[5]. However the onset of akinesia is variable despite using different local anaesthetics and addition of adjuvant [3]. Our clinical experience indicates that it is sometimes necessary when absolute akinesia is required during surgery to augment the block with a second injection of local anaesthetic solution (1–2 ml). Some of the first volume injected for sub-Tenon's block may spill out and the remaining volume may not be enough for akinesia [6], and also the first injection will give time for the effect of hyaluronidase to take place and volume of second injection will spread more to achieve akinesia[7]. Our hypothesis was that a divided injection into the sub-Tenon's space would achieve greater, more consistent and more rapid motor blockade then a single injection using equal volumes and mixtures of local anaesthetic solution.

## Methods

With institutional ethical approval of Cork University hospital and having obtained written informed consent from each, 60 ASA I-III patients undergoing cataract surgery were studied. Exclusion criteria were allergy to any of the drugs administered, impaired mental status, uncontrolled glaucoma, clotting abnormalities, recent surgical procedure on the same eye. This was a single masked, prospective, randomised, control trial. Patients were randomly allocated to one of two groups (group 1: single injection; group 2: two injections) using random number tables. On arrival in the induction room, intravenous access was secured and monitoring with NIBP, ECG, and pulse oximetry (Datex AS3) established. Topical anaesthesia was established by instillation of benoxinate eye drops 0.4% and after 2 minutes an eye speculum inserted. With the eye in neutral position, the conjunctiva was lifted in the infranasal quadrant with the help of Moorfields forceps and using Westcott's scissors a small incision was made in the conjunctiva. A visitec19 gauge Stevens cannula was carefully placed into the sub-Tenon's space. In group 1, 5 ml of local anaesthetic solution comprising 2.5 ml of 2% lignocaine with 1:200,000 adrenaline, 15 IU/ml of Hyaluronidase and 2.5 ml of 0.5% Bupivacaine was injected over thirty seconds. After the injection orbital pressure was applied for two minutes and then movements were measured at 3 and 6 minutes. In group 2, 3 ml of the same anaesthetic solution described above was injected and cannula withdrawn followed by application of orbital pressure for 2 minutes. A further 2 ml of the same anaesthetic solution was injected through the same conjunctival incision with approx two minutes between the two injections and then movements were measured at same time and after 3 minutes that means the first measurement was done at 3 minutes of the first injection and second at 6 minutes of the initial injection. The Intraocular pressures were measured by a hand held tonometer (kowa) prior to injection and six minutes after the first (or sole) injection and compared with pre-injection value. Measurement of movement for the entire study was performed by the same operating surgeon who was unaware of the anaesthetic technique used in all four quadrants (inferior, superior, medial, lateral) using a vernier calliper[8] and scored according to movement, 0-no movement, 1-movement of less then 2 mm and 2-movement of more then 2 mm. Motor function was evaluated at two time intervals, 3 and 6 minutes after the initial injection. In group 1 the Overall movement score was obtained by combining the scores of these four muscles. This score ranged from 0 (no movement) to 8 (complete movement) and was categorised into two groups, akinesia (score 0–4) and no akinesia (score 5–8). Chemosis and subconjuctival haemorrhage were also assessed by surgeons before starting surgery as mild, moderate or severe. Patient's satisfaction was also assessed on scale of unsatisfactory, satisfactory or completely satisfactory from patients in postoperative period.

### Statistical Analysis

Chi-square tests (with Yates continuity correction) were conducted to compare the proportion of patients who did not demonstrate motor block for each of the four quadrants (inferior, medical, superior, and lateral) subconjuctival haemorrhage and chemosis A patient was defined as having no movement if the eye muscle movement score was 0. An overall movement score was obtained by adding the scores of the four quadrants. This score ranged from 0 (no movement) to 8 (complete movement). Chi-square test (with Yates continuity correction) was also employed to compare the percentages of patients who had no akinesia overall between group 1 and 2. Fisher's exact tests were employed when the cell count was less than five. All tests were two-tailed. Significance was determined using P < 0.05. A one-way analysis of covariance (ANCOVA) was performed to examine the differences in post injection intraocular pressures between groups using pre injection intraocular pressure as a covariate. Paired t-tests were conducted to compare the groups in terms of change (pre and post injection) intraocular pressure.

## Results

**Figure 1:**
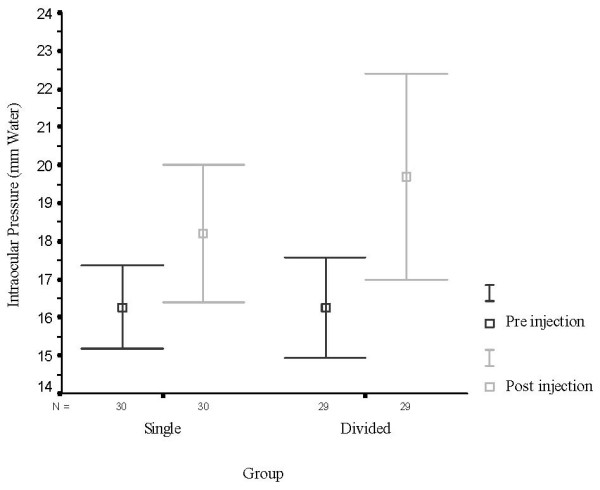
**95% Confidence Intervals of Mean Intraocular Pressure (mm Water) at Pre and Post Injection by Group**. No statistical significance was observed between groups in pre and post injection IOP, although there was statistically significant difference in each group for pre and post IOP.

There was no statistically significant difference in terms of age and gender between groups. Significant differences emerged at 3 minutes between groups for overall movement (P = 0.006), medial (P = 0.039), superior (P = 0.020), and lateral (P = 0.003) quadrants. For these quadrants and overall movement, a significantly greater percentage of patients in group 2 had no akinesia. While for inferior quadrant the percentage of patients with no movement was higher in the divided group but this difference did not reach statistical significance (table 1). At 6 minutes no significant group differences emerged for the overall movement or for any of four quadrants (table 1). No significant difference occurred between groups for levels of haemorrhage, chemosis and patients satisfaction (table 2). Majority of both groups had mild levels of haemorrhage (single 73.3% vs. divided 69%) and chemosis (single70% vs. divided 59%). There was no significant difference in post injection intraocular pressure between groups. Significant changes in intraocular pressure were observed before and after injection for each group. Table 3 gives the means (standard errors) of intraocular pressure at pre and post injection for each group. For each of these groups, the means (SDs) for the paired differences (before and after injection intraocular pressure), and 95% confidence intervals are also presented. Intraocular pressure increased significantly between before and after injection for each group (Fig 1).

**Table 1:** Proportion of patients with no akinesia

	At 3 minutes			At 6 minutes		
	
	**Group1** (n = 30) n (%)^1^	**Group 2** (n = 30) n (%)	**P-value**	**Group1** (n = 30) n (%)^1^	**Group 2** (n = 30) n (%)	**P-value**
Overall movement	10 (33.3)	21 (72.4) *	0.006	1 (3.3)	2 (6.9) *	0.612

Inferior quadrant	7 (23.3)	13 (44.8)	0.142	3 (10.0)	2 (6.9) *	1.000

Medial quadrant	5 (16.7)	13 (44.8) *	0.039	2 (6.7)	2 (6.9) *	1.000

Superior quadrant	2 (6.7)	10 (34.5) *	0.020	0	0 *	1.000

Lateral quadrant	10 (33.3)	22 (75.9) *	0.003	4 (13.3)	8 (27.6) *	0.209

^1 ^number (%) of patients who had no akinesia, * p < 0.05 compared to group

**Table 2:** Levels of Haemorrhage, Chemosis, and Patient Satisfaction by Group

	**Single **(n = 30) n (%)	**Divided **(n = 29) n (%)	**P-value**
**Haemorrhage**			
Mild	22 (73.3)	20 (69.0)	0.934
Moderate	8 (26.7)	9 (31.0)	

**Chemosis**			0.522

Mild	21 (70.0)	17 (58.6)	
Moderate	9 (30.0)	12 (41.4)	

**Patient Satisfaction**			0.914
Satisfied	12 (40.0)	12 (41.4)	
Complete satisfied	18 (60.0)	17 (58.6)	

**Table 3:** Means (SE) of Intraocular Pressure

	**Mean (SE)**	**95% CI**
Single (n = 30)		
Pre- injection	16.3 (0.5)	
Post- injection	18.2 (0.9)	
Paired Difference	-1.9 (0.9)	-3.7 – -0.1

Divided (n = 30)		
Pre- injection	16.2 (0.6)	
Post- injection	19.7 (1.3)	
Paired Difference	-3.5 (1.3)	-6.2 – -0.7

*Note*: SE = standard error or mean.

## Discussion

An ideal eye block technique would provide globe analgesia, akinesia, and absence of pressure on the globe, minimal injectate volume, without serious complications [9]. The sub-Tenon's approach to ocular local anaesthesia differs from retrobulbar and peribulbar methods in that it is performed using blunt instrumentation and direct visualisation and therefore relatively safer [10]. The delivery of sub-Tenon's anaesthetic is effective and reliable in producing both akinesia and anaesthesia. Ultrasound monitoring clearly demonstrated leakage of solution out of Tenon's space to the intraconal space [11]. Sub-Tenon's anaesthesia appears to be a more effective method of anaesthesia than the peribulbar method[12]. Our clinical experience indicated that it is sometimes necessary to augment the block with 1–2 ml of local anaesthetic solution to achieve absolute akinesia, which also was done in several other trials[9,13]. There is a valve effect and some of the volume injected spills out of the incision made and so more volume is needed to have enough volume for the desired effect [6]. It may be after the first injection, hyaluronidase hydrolyses part of intracellular matrix that maintain tissue integrity and the second injection would easily disperse more extensively around the orbit and help in achieving akinesia. [7]. We used the mixture of lignocaine with 1/200,000 adrenaline, Bupivacaine and Hyaluronidase. Previous studies indicate adding Hyaluronidase resulted in a greater degree of akinesia [4,14,15]. We used 5 ml volume as volumes 4 ml and less required additional facial nerve block for persistent orbicularis tone[9]. In our study we did not find any benefit of two injections and single injection was superior to provide akinesia of ocular muscles, similar results were also observed when one versus two injection were tried in peribulbar anaesthesia [13]. In our study there was good akinesia at 6 min in both groups, where as in Stevens study only 27 patients out of fifty had complete akinesia at fifteen minutes [4]. Greenbaum reported good akinesia within 1 minute of block but he used 4% lignocaine solution and 0.75% bupivacaine 50:50 mixture [16]. Chemosis was present in both groups but was not statistically significant. It is one of the recognised side effects of sub-Tenon's but little practice is needed to deliver the anaesthetic solution to posterior sub-Tenon's space and not to the anterior subconjuctival space[5]. Mild to moderate haemorrhage was noted in both groups but there was no statistically significant difference among groups, Greenbaum advocate cauterisation before making a buttonhole to lower the incidence of haemorrhage [16]. There was no statistically significant difference in IOP between the two groups. These results were similar to a previous study where volume of 3 ml to 5 ml was compared for IOP [17].

## Conclusion

Single injection of local anaesthesia for sub-Tenon's block with mixture of lignocaine with adrenaline, bupivacaine and hyaluronidase was found to be superior to provide akinesia of ocular muscles at three minutes compared to divided dose given by two injections and at six minutes no difference was found and hence there is no advantage of giving sub-Tenon's block by two injections over single injection. No statistical difference was found in degree of subconjuctival haemorrhage, Chemosis, Patients satisfaction and intraocular pressure between groups

## Competing interests

The authors declare that they have no competing interests.

## Authors' contributions

EK, JM, JMcA, GS were involved acquisition, analysis and interpretation of data, drafting the manuscript, revising it critically for important intellectual content and have given final approval of the version to be published.

## Pre-publication history

The pre-publication history for this paper can be accessed here:


